# The Relationship Between Climate Change Issue Engagement, Connection to Nature and Mental Wellbeing

**DOI:** 10.3389/fpubh.2022.790578

**Published:** 2022-05-09

**Authors:** Matt Whelan, Shahin Rahimi-Golkhandan, Eric Brymer

**Affiliations:** ^1^School of Psychology and Wellbeing, Faculty of Health, Engineering and Sciences, University of Southern Queensland, Toowoomba, QLD, Australia; ^2^Faculty of Health, Southern Cross University, Bilinga, QLD, Australia

**Keywords:** climate change, wellbeing, nature relatedness, pro-environmental behavior, nature connection/intimacy

## Abstract

As the threat of climate change becomes increasingly prevalent for people in both the developed and developing world, the impact of climate change on mental wellbeing has become a crucial area of research. In addition to the direct, indirect, and psychosocial impacts of climate change on mental wellbeing, there is also a question of how climate change driven changes to the environment will influence the well-established positive relationship between connection to nature and mental wellbeing. The aim of this study was to investigate the relationship between climate change issue engagement, connection to nature, and mental wellbeing in English speaking adults over 18 years of age. This study examined the average levels of connection to nature and mental wellbeing in people with different levels of climate change issue engagement, and evaluated whether a person's level of climate change issue engagement uniquely predicted mental wellbeing. The study corroborated positive relationships between wellbeing and various aspects of relatedness to nature in the overall sample. The strength of these relationships, however, depended on the level of climate change issue engagement. More specifically, the level of engagement is inversely linked to mental wellbeing, such that the lower the level of engagement, generally the higher is wellbeing.

## Introduction

The last decade has been witness to considerable research into the mental health benefits of interacting with nature (1–3). In general, studies have consistently found a positive relationship between high levels of nature relatedness (NR) and mental wellbeing (MWB). For example, Martyn and Brymer (4) found that high NR was associated with low anxiety and that physical familiarity with nature was more likely to predict low levels of anxiety. Lawton et al. (5) also found that familiarity with outdoor environments, as measured by physical activity choice, predicted lower anxiety. To an extent Dean et al. (6) echoed these findings, however they also indicated that self-identification with nature might impact negatively on mental health. They suggested that active engagement with pro-environmental behavior and awareness about how actions impact the environment might be associated with negative mental health impacts. As the threat of climate change becomes increasingly prevalent, the profound changes to temperature and the increased incidence of extreme weather events are expected to have an increasingly adverse impact on nature and human systems (7).

Climate change is already having an adverse impact on MWB and the trajectory is rapidly accelerating (8). According to Doherty and Clayton (9), negative mental health outcomes might increase with awareness of issues around climate change. In this case a strong NR could potentially negatively impact mental wellbeing, rather than promote it. In recognition of this, one potential factor that could diminish or reverse the positive relationship between NR and mental wellbeing is an individual's climate change issue engagement (CCIE). According to Lorenzoni et al. (10), CCIE is a sense of “connection with the issue of climate change that concurrently encompasses cognitive, affective, and behavioral aspects” (p. 446). Rather than just an attitude or concern, CCIE also encompasses the motivation, willingness, and ability to take (or already taking) personal and/or political action (11). The relationship between NR, climate change engagement, and mental health is complex. From a mental health perspective, understanding the mechanisms behind this relationship is vital for protecting and promoting mental health as the world comes to terms with the impact of global climate change.

Although the direct (e.g., responses to heatwaves or natural disasters) and psychosocial ramifications of climate change have received considerable research attention, research examining the indirect impact of climate change is still limited (12). There is also limited research on the indirect impact (e.g., thinking about climate change impacts) of climate change on the well-established positive association between NR and MWB (6). That is, while there is reliable evidence that reduced ill-health is associated with enjoyment of nature, research suggests that people who incorporate nature into their sense of self may perceive the climate change driven harm to nature as harm done to themselves. Consequently, people with high NR might be more susceptible to the indirect impact of climate change and reduced mental health (6, 9).

Perception is an important factor influencing the relationship between NR and MWB. In particular, the pro-environmental aspects of NR can actually be harmful to MWB when an individual feels their pro-environmental behavior is dedicated to an environmental goal that is perceived to be unattainable (13). With the increasing threat of climate change and insufficient response by some of the wealthiest nations, it is plausible that an individual with high levels of CCIE might experience lower average levels of MWB. In addition, as NR is associated with behaviors aimed to help address climate change, it is also plausible that CCIE might influence the well-established relationship between NR and MWB. However, these scenarios are yet to be explicitly examined (12). The aim of this study was 2-fold: (a) examine whether the relationship between NR and MWB depends on the level of CCIE? (b) determine whether CCIE uniquely predicts MWB, over and above the impact of age and NR?

## Methods

### Participants

The study consisted of a total of 407 people aged between 18 and 79 years (M = 35.14, SD = 15.71). One hundred and seventy-two participants identified as male (42.3%); 231 as female (56.8%); and four did not specify (1%). Forty-one different nationalities were identified by participants, including: 189 who identified their nationality as Australian (46.4%); 84 as American (20.6%); 26 as British (6.4%); 13 as Canadian (3.2%); 12 as German (2.9%); six as Norwegian (1.5%); five as Danish (1.2%); five as Scottish (1.2%); four as Dutch (1%); four as Finnish (1%); four as Indian (1%); four as New Zealander (1%); 49 from 29 other nationalities (12.1%); and two who did not specify (0.5%).

### Materials

Participants completed an online survey consisting of seven demographic questions and a series of questionnaires assessing CTN, CCIE, and MWB. The demographic questionnaire consisted of questions relating to participants' age, gender, income, nationality, level of education, and political orientation.

#### Connection to Nature

The Nature Relatedness Scale is a 21-item scale (14) used to measure individual experiences of connection to nature (CTN) (15). The scale consists of three subscales: NR-Self, NR-Perspective, and NR-Experience. NR-self is an 8-item subscale used to measure how much an individual identifies with nature (e.g. “My connection to nature and the environment is a part of my spirituality”). NR-Perspective is a 7-item subscale used to measure how concerned an individual is about the impact of human actions on the environment and living things (e.g. “I think a lot about the suffering of animals”). NR Experience is a 6-item subscale used to measure an individual's level comfort with and desire to be out in nature (e.g. “I enjoy being outdoors, even in unpleasant weather”). Items were rated on a five-point Likert-type scale from 1 (disagree strongly) to 5 (agree strongly). The total score was calculated by averaging all 21 items (after reverse scoring appropriate items), with higher scores indicating a stronger connection to nature (4, 5). A Cronbach's alpha of 0.87 for the total scale, 0.84 NR-Self items, 0.66 for NR-Perspective items, and 0.80 NR-Experience were reported in the original study (14). Although NR-Perspective's was <0.70, values between 0.60 and 0.70 can be deemed in the lower limit of acceptability (16).

#### Climate Change Issue Engagement

The Global Warming's Six Americas questionnaire (GWSA) is a psychographic audience segmentation tool used to measure climate change issue engagement (CCIE) (17). The questionnaire identifies participants as belonging to one of six distinct subgroups, which are distinguishable based on their climate change concern, beliefs, issue involvement, political and consumer advocacy behaviors, preferred societal responses, and underlying barriers to action. The six segments range across a spectrum of climate change concern and issue engagement, with segments most accepting and most rejecting of climate change science at either end of a continuum. At one end of the CCIE continuum is the Alarmed segment, which consists of people most certain climate change is happening, most concerned about it, and most active in their actions to mitigate it. At the other end is the Dismissive segment, which consists of people most certain climate change is not happening, unconcerned about it, and opposes action to mitigate it (11). Between the Alarmed and Dismissive segments are those that devote less thought to climate change and are less certain of their views (18). Studies examining each of the six segments are used by climate change communicators—government agencies, non-governmental organizations, and media organizations—to tailor communication and educational content to better engage specific target audiences (19). The original six segments were identified using linear discriminant functions and the 15-item instrument correctly classifies 84% of the sample when compared with the original Latent Class Analysis results (17). A subsequent study reported a Cronbach alpha of 0.87 (11). While initially designed for market segmentation the CCIE has proven to be a valid research tool for measuring climate change issue engagement (20, 21). For a detailed explanation of the six audience segments, see (19).

#### Mental Wellbeing

The Warwick Edinburgh Mental Wellbeing Scale was used to measure both hedonic and eudaimonic aspects of MWB including: “psychological functioning, life satisfaction, and ability to develop and maintain mutually beneficial relationships” [(22), p. 2]. The scale consists of 14 positively worded statements covering subjective wellbeing and psychological functioning (e.g. “I've been feeling good about myself”). Items were rated on a five-point Likert-type scale from 1 (none of the time) to 5 (all the time). The total score was calculated by summing all 14 items, ranging from 14 to 70, with higher scores indicating a higher level of MWB. A Cronbach's alpha of 0.89 was reported in the original study (22).

### Procedure

Following ethics approval from the human research committee at the Australian College of Applied Psychology, participants were recruited via three methods: (1) student cohorts via SONA; (2) social media websites Facebook, Twitter, and Reddit; and (3) potential participants who contacted the researchers were informed by email. Participants recruited through SONA received course credit for participation. Participants recruited through Facebook, Twitter, and Reddit were provided with a link to the online survey. Participants recruited through email were provided with basic information about the study and a link to the online survey. To ensure that participants recruited through different methods were provided with the same information, the Participant Information Statement was added to the first page of the online survey. Participants were informed that the study was an online survey investigating the relationship between attitudes toward climate change, CTN, and MWB. Participants were informed that the survey consisted of non-identifiable demographic questions, followed by items about how strongly they agreed or disagreed with a statement, and were given instructions regarding their participation and confidentiality. Participants were advised that participation was voluntary and that clicking “Yes” at the bottom of the Consent Statement was an indication of their consent. Participants were also advised that because their responses were not attached to their identity, their responses could not be removed once they had submitted their survey. All online survey data were collected using “Qualtrics” and converted to an IBM SPSS Statistics Version 25 (23) file for analysis.

## Results

### Demographics

The average MWB of the participants (47.1) was slightly below the established norm (51.0) for adults on the Warwick-Edinburgh MWB Scale. This was also reflected in the average MWB for men (47.54) and women (46.71) in the study, which were both below established norms for men (51.3) and women (50.3) (Tennant et al., 2007). There was no statistically significant difference between the average MWB scores of males and females in the current study (*p* = 0.42).

### CCIE Group Comparisons

**Figure 1:**
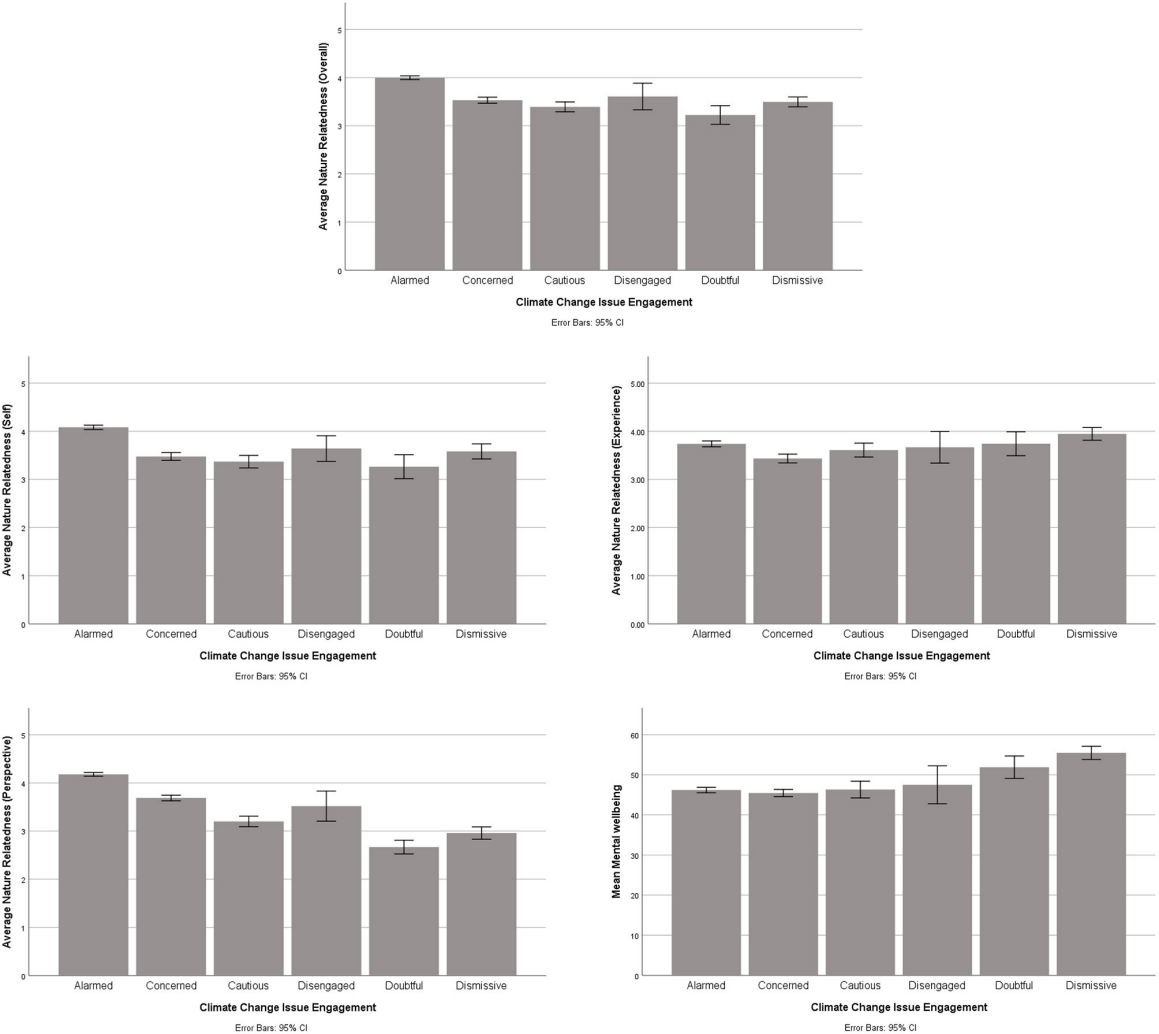
Average levels of related to nature and wellbeing in CCIE groups.

We compared the average MWB as well as NR between participants in each of the six groups of CCIE. Given the small sample size in the disengaged (*n* = 8) and doubtful (*n* = 19) groups, any test of normality would have produced unreliable results. Thus, for each variable, we compared the groups using the Kruskal-Wallis test. Figure 1 includes bar charts showing average scores of MWB and NR in each of the six groups.

The Kruskal-Wallis test was significant for the overall NR (*p* < 0.001). *Post-hoc* analyses indicated the “alarmed” group had significantly higher levels of relatedness to nature than the “concerned,” “cautious,” “doubtful,” and “dismissive” groups (*p* < 0.001). The overall test was also significant for NR-Self (*p* < 0.001). In a similar vein to the overall relatedness to nature, the “alarmed” group reported significantly higher levels of NR-self than the “concerned” (*p* < 0.001), “cautious” (*p* < 0.001), “doubtful” (*p* = 0.007) and “dismissive” groups (*p* = 0.024). For NR-Perspective, the overall test was significant (*p* < 0.001). Once again, the “alarmed” group had significantly higher scores than the “concerned,” “cautious,” “doubtful,” and “dismissive” groups (*p* < 0.001). Moreover, NR-Perspective was also significantly higher in the “concerned” group than the cautious” (*p* = 0.042), “doubtful” (*p* < 0.001) and “dismissive” groups (*p* = 0.002). The CCIE groups, however, were not significantly different to each other on NR-experience (*p* >.064).

For MWB, we identified a significant overall effect (*p* < 0.001). The “dismissive” group reported a significantly higher average wellbeing than the “alarmed” (*p* < 0.001), “concerned” (*p* < 0.001), and “cautious” (*p* = 0.026) groups.

### Correlational Analyses

We examined the relationship between MWB and each subscale of the Nature Relatedness Scale (i.e., NR-Self, NR-Experience, NR-Perspective). Given that all the NR subscales showed negative skew and violated the assumption of normality, bivariate relationships were examined using Spearman's ρ. Cohen's guidelines were used to interpret the strength of each relationship.

There were significant weak positive relationships between wellbeing and NR-Self [ρ (*N* = 390) = 0.20, *p* < 0.001], and wellbeing and NR-Experience [ρ (*N* = 391) = 0.25, *p* < 0.001]. However, no statistically significant relationship was found between wellbeing and NR-Perspective [ρ (*N* = 391) = −0.08, *p* = 0.11].

**Figure 2:**
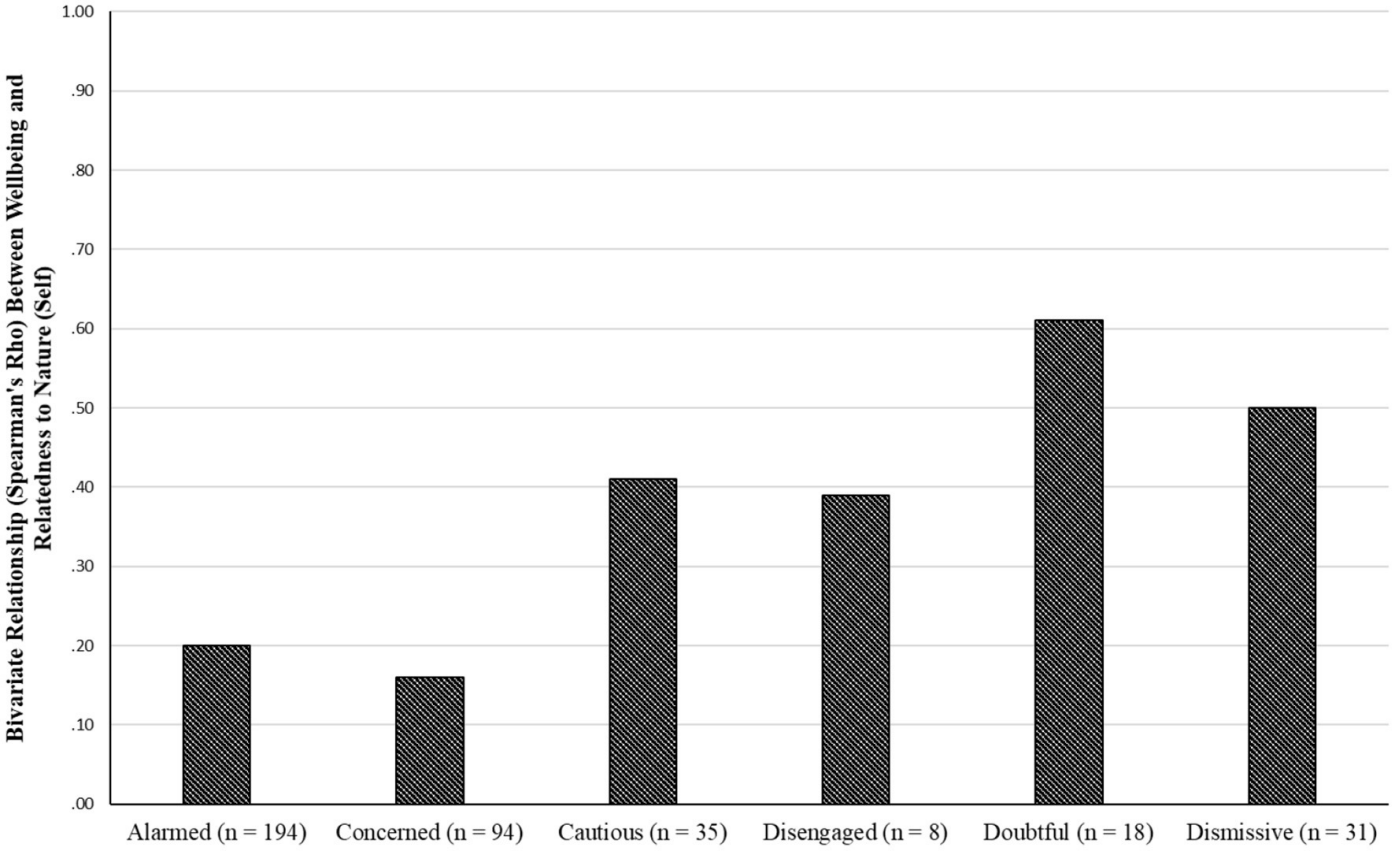
Bivariate relationship between wellbeing and NR-self.

We also examined these three bivariate relationships (using Spearman's ρ) in each of the six group of participants, as defined by their level of CCIE (i.e., alarmed, concerned, cautious, disengaged, doubtful, and dismissive). Figure 2, which presents the relationship between wellbeing and NR-Self, shows that the significant positive relationship that was observed for the overall group, occurred in each level of CCIE. However, the relationship did not reach statistical significance among the “concerned” and “disengaged” participants. Although the relationship remained weak in the “alarmed” group, it was strong on the other end of the continuum, for both the “doubtful” and “dismissive” participants. This indicates that wellbeing is more tightly linked to NR-Self among these two groups.

**Figure 3:**
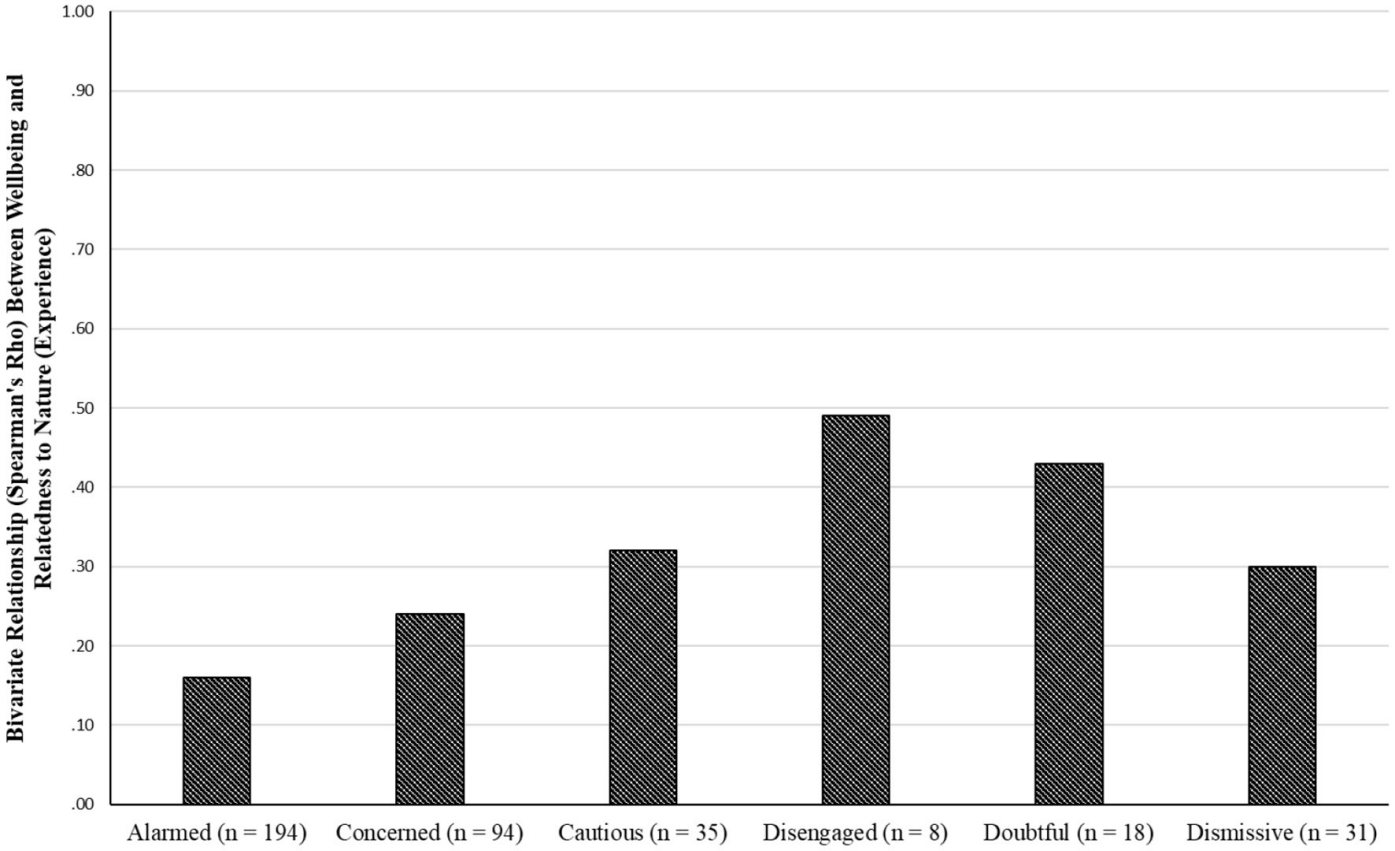
Bivariate relationship between wellbeing and NR-experience.

Figure 3 depicts the relationship between wellbeing and NR-Experience. Although the relationship remained positive in all groups (as was the case with the overall group), the only two significant relationships were observed in the “alarmed” and the “concerned” groups. However, this could be due to their noticeably larger samples sizes compared to the other groups, who indeed showed stronger, yet non-significant relationships. For instance, Spearman's ρ in both the “disengaged” and “doubtful” groups was moderate to strong (over 0.40), but it did not reach statistical significance. There were <20 participants in either of these two groups.

**Figure 4:**
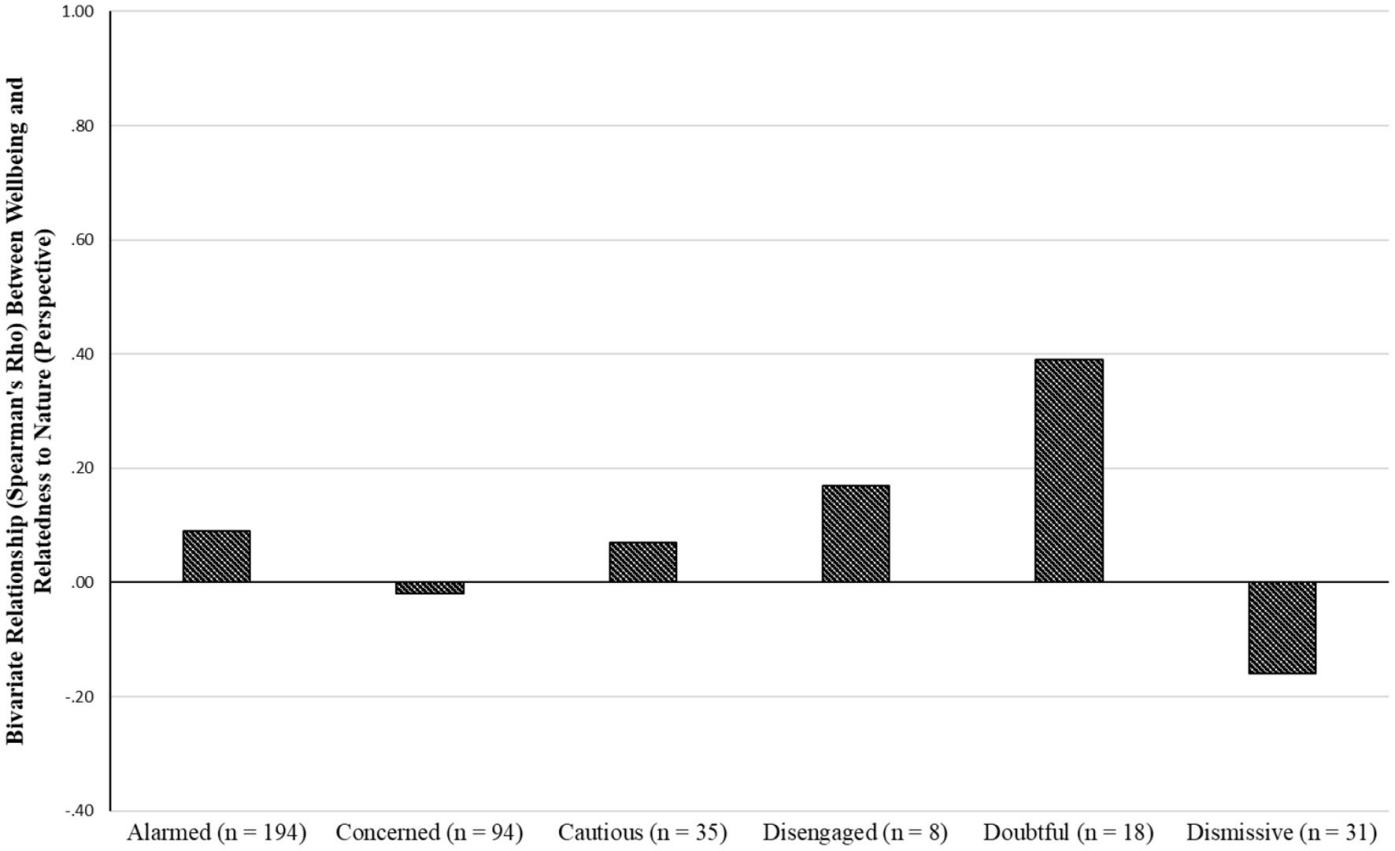
bivariate relationship between wellbeing and NR-perspective.

Figure 4 shows that the relationship between wellbeing and NR-Perspective was not significant in any of the six groups. All groups, except for the “doubtful”, showed weak relationships. Interestingly, however, the direction of the relationship was negative in the “dismissive” group. Although the strength of the relationship was moderate in the “dismissive” group, it, once again, failed to reach statistical significance. As noted earlier, we suggest this non-significant finding could be linked to the small size of this group.

### Regression Analysis

To examine whether CCIE uniquely predicts MWB, we ran a hierarchical regression analysis with age and the three subscales of the Nature Relatedness scale as predictors in step 1, and then in step 2, the CCIE was added as the last predictor. As noted earlier, scores on the CCIE variable ranged from 1 = “alarmed” to 6 = “dismissive”. Two participants who had a standardized residual greater than three were deemed outliers and were removed from the regression analysis.

The overall model was significant in step 1 and explained 16.1% of wellbeing variance in the population, *F*_(4,358)_ = 18.34, *p* < 0.001. Age (β = 0.18, *p* < 0.001), NR-Self (β = 0.27, *p* < 0.001) and NR-Perspective (β = −0.27, *p* < 0.001) significantly predicted 2.99, 3.13% and 4.58% of unique variance in wellbeing. The regression coefficient for NR-Experience was not significantly different to zero in the population (*p* = 0.076).

The model remained significant after adding CCIE in step 2, *F*_(5,357)_ = 16.89, *p* < 0.001, and explained 18% of variance in wellbeing in the population. CCIE was a significant predictor (β = 0.21, *p* = 0.002) and explained 2.13% of unique variance in MWB. The introduction of CCIE to the model in step 2 resulted in age no longer being a significant predictor of wellbeing (*p* = 0.059). NR-Self (β = 0.30, *p* < 0.001) and NR-Perspective (β = −0.16, *p* = 0.022) significantly predicted 3.80 and 1.21% of wellbeing, respectively.

## Discussion

### Overview

The aim of this study was 2-fold: (a) to determine whether the relationship between MWB and NR depends on the level of engagement with the issue of climate change, and (b) to examine whether CCIE can uniquely predict MWB, over and above the impact of NR and age.

First, our findings indicate those with higher levels of CCIE (i.e., the “alarmed” and “concerned” groups) generally have higher levels of NR, as evidenced by their scores on NR-Self and NR-Perspective. The impact of CCIE on NR was more evident in the “alarmed” group who reported significantly higher scores on overall relatedness to nature. These findings are in line with previous research indicating that those with a greater awareness of climate change and its consequences are more likely to report higher levels of NR (6).

The finding that the Dismissive segment averaged significantly higher levels of MWB than the Cautious, Concerned, and Alarmed segments provide novel insights in both the potentially protective factors of low climate-engagement and the potentially detrimental impact of increasingly high climate-engagement. However, it should be noted that the Cautious, Concerned, and Alarmed segments were well within one standard deviation of the average levels of MWB for the whole sample. Although the Warwick-Edinburgh MWB Scale (22) is not designed to identify individuals with exceptionally low or high positive mental health, the results provide new insights into the each of the six CCIE segments, as MWB has not previously been measured in any studies that have used the Global Warming's Six Americas audience segmentation tool (17).

It is possible that the below average MWB found in the Cautious, Concerned, and Alarmed segments in this study could be attributed to anxiety from the threat or anticipation of harm from climate change, as found in Gifford and Gifford (24). Similarly, it is possible that the below average MWB in the Cautious, Concerned, and Alarmed segments could be the result of a high prevalence of ecological grief in the sample (25). Believed to be more prevalent among those with a strong NR, an above average rate of ecological grief in these segments is plausible, as those with a higher level of engagement with the issue of climate change were more likely (in the current study) to report higher overall scores on the Nature Relatedness Scale.

Second, we showed that the strength and significance of the relationship between NR and MWB depends on the level of CCIE. More specifically, the groups with the least level of engagement with climate change (i.e., “doubtful” and “dismissive participants”) reported the strongest positive relationships between wellbeing and NR-Self, compared to the other groups. The results for the other two sub-scales of the Nature Relatedness Scale were inconclusive. For instance, even though the relationship between NR-experience and wellbeing was only statistically significant in the “alarmed” and the “concerned” groups, the strength of the relationship was higher among the “disengaged” and “doubtful” groups. We believe the non-significant relationships in these two groups is due to their smaller sample sizes, though it is also possible that this may be because the dismissive and doubtful groups do not link climate change with human actions. The overall, qualitative trend, however, is similar to that of NR-Self, in that, the relationship between MWB and an aspect of relatedness to nature is stronger in those with less concern about climate change.

Finally, regression analysis indicated that CCIE is a significant predictor of MWB, over and above the impact of age and NR. Our initial model incorporated age and three sub-scales of the Nature Relatedness Scale as predictors of MWB. Age, NR-Self, and NR-Perspective were significant predictors of wellbeing in the initial model. However, the addition of CCIE in the second model made age a non-significant predictor of wellbeing. Instead, CCIE predicted over 2% of unique variance in MWB. This finding underscores the importance of paying attention to CCIE as an important contributor to wellbeing. These findings point to the need for greater attention to be focused on the impact of CCIE on psychological outcomes, and support previous literature, such as Maibach et al. (17), who reported that high levels of CCIE was associated with fear, sadness, anger, and disgust; as well as Randall (26), who cited symptoms of grieving and loss among people who had recently learned of the ramifications of climate change and the lifestyle changes needed to minimize their own carbon footprint. Taken together, these findings support Doherty and Clayton (9), who suggested that the indirect impact of observing climate change-related environmental changes around the globe will have a negative impact on MWB. Hayes et al. (8) recognized this impact and determined that as risks increase, direct and indirect impacts become progressively more prevalent suggesting that great coordination, based on the provision of hope, is required to counteract the mental health impacts of climate change.

In support of the U.S National Wildlife Federation (27), one potential explanation for the relationship between CCIE and mental MWB is the added perception that human behavior has contributed to climate change. Normally observed in people who have witnessed a technological disaster, the perception that human behavior caused a disaster can result in strong emotions, such as anger and distrust, over-and-above symptoms common in disasters where human behavior is not perceived to be at fault. The indirect impact of climate change has been linked with a reduction in cultural and personal identity (28), personal security, sense of place (25), belonging (29), and increased levels of anxiety (24). However, the results in this study suggest that the indirect impact on MWB may potentially warrant more investigation into symptoms, treatments, and predictors of psychoterratic (emotions felt in relation to earth) mental illnesses, such as eco-anxiety and ecological grief (30). Indeed, as climate change-related natural disasters become more frequent and people become increasingly climate-engaged (31), the prevalence of the population that could be segmented in the most climate-engaged CCIE segments is likely to increase considerably (32).

Overall, results suggest that people in the most climate-engaged “Alarmed” segment experience lower levels of MWB, are more connected to nature, identify more with nature, and are more concerned about humans' impact on the environment than the less climate-engaged segments. These findings are a novel contribution to the literature investigating the indirect impact of climate change; the link between CCIE, MWB, and NR; as well as the Global Warming's Six Americas project, which endeavors to gain more insight into the attitudinal and behavioral characteristics of each of the six audience segments in order to best equip government and non-governmental organizations to engage and inform people about the issue of climate change.

## Limitations and Future Directions

One of the limitations of the current study was that the sample consisted of participants who completed an online survey and were fluent in English. As such, the study was not representative of people who do not have access to the Internet and people who are not fluent in English. In addition, many of the participants were recruited using social media platforms. Thus, there may also have been an over representation of people who use social media. Thirdly, the low number of people in the Disengaged segment (*n* = 8) could suggest that people who did not have strong beliefs about climate change did not participate in the study. As such, the results that compared the Disengaged segment should be taken with some caution. Fourth, the study consisted of more than double the number of people with a liberal political orientation (*n* = 119) than people with a conservative political orientation (*n* = 52). Thus, as political orientation is a predictor of CCIE, it is possible that the sample had an over-representation of people with high levels of CCIE than in the population. Consequently, future research may reveal more insights into the relationship between MWB, NR, and CCIE in a wider representation of the adult population.

### Implications

This study not only adds to our understanding of the relationship between MWB and NR, it also sheds light on the levels of NR and MWB of people in each of the Global Warming's Six Americas audience segments (17). One of the important implications of this study is that CCIE likely plays a role in the MWB of people. While the growing interest regarding the impact of climate change on mental health (9, 33, 34) was a catalyst for this study, the findings support the assertion that the indirect impact of climate change on MWB has important implications for both mental health practitioners and policy makers. In addition, the results should also encourage future research in the factors contributing to the MWB of people with high CCIE. Moreover, the finding that there was no association between NR-Perspective and MWB provides more context to the suggestion that CCIE may influence the established positive association between NR and MWB (9). The apparent distinction between pro-environmental beliefs and pro-environmental behavior in determining the relationship between CCIE, NR, and MWB is one that should encourage future research to determine what factors account for the distinction, and what protective factors and vulnerabilities can alter its influence.

## Conclusion

In conclusion, the study revealed that people with high CCIE were more likely to report lower levels of MWB, and higher average levels of NR-Total, NR-Self, and NR-Perspective. The study also revealed that the relationship between NR and MWB is more likely to be stronger among those with lower levels of CCIE (i.e., “disengaged,” “doubtful,” and “dismissive” groups). We also showed that CCIE uniquely predicts MWB. As policymakers attempt to engage with people at varying levels of climate engagement, targeted campaigns to these discrete distinguishable segments of the population are likely to be far more effective than campaigns designed to inform a larger section of the audience (18). An improved understanding of the six audience segments might also help information campaigns become more effective in informing populations about the threat of climate change and to encourage acceptance for the changes necessary to prevent global temperatures exceeding CO_2_ above pre-industrial global average surface temperature.

## Data Availability Statement

The raw data supporting the conclusions of this article will be made available by the authors, without undue reservation.

## Ethics Statement

The studies involving human participants were reviewed and approved by Navitas Professional Ethics Committee. The patients/participants provided their written informed consent to participate in this study.

## Author Contributions

MW: conception, design, implementation, and analysis. SR-G: analysis and writing. EB: conception, design, analysis, and writing. All authors contributed to the article and approved the submitted version.

## Funding

Funds will be received from Australian College of Applied Psychology on acceptance.

## Conflict of Interest

The authors declare that the research was conducted in the absence of any commercial or financial relationships that could be construed as a potential conflict of interest.

## Publisher's Note

All claims expressed in this article are solely those of the authors and do not necessarily represent those of their affiliated organizations, or those of the publisher, the editors and the reviewers. Any product that may be evaluated in this article, or claim that may be made by its manufacturer, is not guaranteed or endorsed by the publisher.
